# A Giant Retropharyngeal Lipoma: A Case Report and Review of Literature

**DOI:** 10.7759/cureus.29022

**Published:** 2022-09-11

**Authors:** Rajaa A Alnami, Somaya M Saabi, Rwan A Mossery, Bushra A Alnami, Mohd Al Ghadeeb

**Affiliations:** 1 General Practice, Jazan University, Jazan, SAU; 2 Radiology, King Fahad Hospital, Hofuf, SAU

**Keywords:** case report, hoarse voice, dysphagia, retropharyngeal mass, lipoma

## Abstract

Lipoma is a common benign soft tissue lesion that can virtually develop anywhere in the body. However, the retropharyngeal space is an extremely rare location for lipoma. We report the case of a 48-year-old man who presented with progressive dysphagia of 3 months duration. It was associated with a weight loss of 6 kg. There was no history of cough, regurgitation, or heartburn. He was a heavy smoker, but he denied consuming alcohol. On examination, the patient was noted to have a hoarse voice. Examination of the oral cavity revealed a bulge in the posterior pharyngeal wall with intact and smooth overlying mucosa. Examination of the neurological system revealed normal findings. The patient was referred to undergo a computed tomography of the neck, which demonstrated a well-defined homogeneous lesion with fat density in the retropharyngeal space. Subsequently, magnetic resonance imaging revealed a well-encapsulated midline retropharyngeal mass, measuring 4.6 x 10.2 x 13.8 cm, filling the retropharyngeal space and extending from the C2 vertebra superiorly to the inferior border of C7 inferiorly. The mass has a high signal intensity on T1- and T2-weighted images with complete suppression of the signal on the fat-saturated sequences, likely representing a retropharyngeal lipoma. The patient underwent surgical resection of the tumor by the lateral cervical approach. Histopathological examination showed lobules of mature adipose tissue, representing a lipoma. Retropharyngeal space is a very rare location of lipoma. The case highlights the importance of considering retropharyngeal lesions when encountering a patient with progressive dysphagia.

## Introduction

Lipoma is the most common benign mesenchymal tumor and can virtually develop anywhere in the body [1]. It is estimated that 13% of lipomas occur in the head and neck region [1]. The most frequent location of the lipoma of the head and neck region is in the subcutaneous space of the posterior triangle [2]. However, it is rare for a lipoma to occur in the deep spaces of the neck. Primary tumors of the retropharyngeal space are exceedingly rare [2,3]. There has been a limited number of reports in the literature on retropharyngeal lipomas [2]. This tumor can present with a myriad of clinical symptoms related to pressure effect on adjacent structures, including dysphagia, dysphonia, and dyspnea. Here, we report the case of a giant retropharyngeal lipoma in a middle-aged man presenting with progressive dysphagia who has undergone successful surgical resection of the tumor.

## Case presentation

A 48-year-old man attended the primary healthcare center with a complaint of difficulty in swallowing for 3 months duration. Initially, he experienced difficulty swallowing certain solid foods. However, his difficulty in swallowing progressed to involve both solids and liquids. The difficulty in swallowing was associated with an unintentional weight loss of 6 kg in the last month. There was no history of cough, regurgitation, or heartburn. Furthermore, no history of painful swallowing or shortness of breath was given. Regarding his past medical history, he had a 20-year history of well-controlled hypertension, diabetes mellitus, and dyslipidemia. No history of peptic ulcer disease was given. The family history was remarkable for systemic lupus erythematosus in his two siblings. He was a heavy smoker (25 pack-years). He never consumed alcohol or used recreational drugs.

On examination, the patient was alert, conscious, and oriented. The vital signs were within the normal range. He had a body mass index of 27.5 kg/m^2^. He was noted to have a hoarse voice. Examination of the oral cavity revealed a bulge in the posterior pharyngeal wall with intact and smooth overlying mucosa. On neurologic examination, the patient had normal gag reflex and normal cranial nerves examination. There was no impairment of the motor or sensory function. No lymphadenopathy or palpable neck masses were observed. Laboratory investigations, including hematological and biochemical profiles, were normal. During the laryngoscopy examination, a marked protrusion of the posterior pharyngeal wall with narrowing of the hypopharynx was noted. The larynx appeared normal.

**Figure 1:**
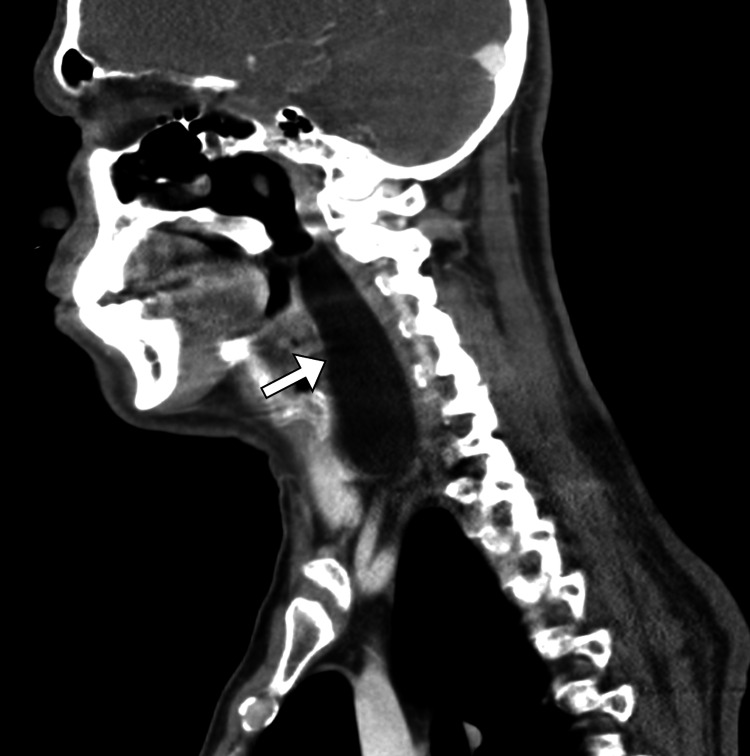
Sagittal CT image of the neck showing a well-defined retropharyngeal lesion with fat density (arrow). CT: computed tomography.

**Figure 2:**
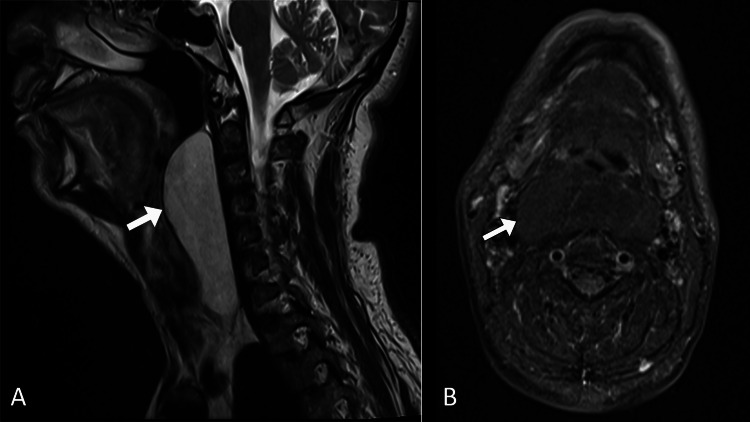
MR images of the neck in the sagittal (A) and axial (B) planes showing a well-defined retropharyngeal lesion (arrows) with a high signal intensity on T2-weighted image (A) and complete suppression on fat-suppressed sequence (B). MR: magnetic resonance.

The patient was referred to undergo a computed tomography (CT) scan of the neck, which demonstrated a well-defined homogeneous lesion with fat density in the retropharyngeal space (Figure 1). Subsequently, the patient underwent a magnetic resonance imaging (MRI) scan for further characterization and evaluation. The MRI scan revealed a well-encapsulated midline retropharyngeal mass, measuring 4.6 x 10.2 x 13.8 cm, filling the retropharyngeal space and extending from the superior border of the C2 vertebra to the inferior border of the C7 vertebra. The mass had a high signal intensity on T1- and T2-weighted images with complete suppression of the signal on the fat-saturated sequences. The mass has homogeneous signal intensity with no evidence of thick septations. It demonstrated no post-contrast enhancement nor restriction of diffusion. The mass was abutting the superior palate and caused significant narrowing of the oropharynx. The superior trachea and hypopharynx were normal. Furthermore, the common carotid arteries and internal jugular veins were displaced posterolaterally (Figure 2). Such findings were consistent with a retropharyngeal lipoma.

**Figure 3:**
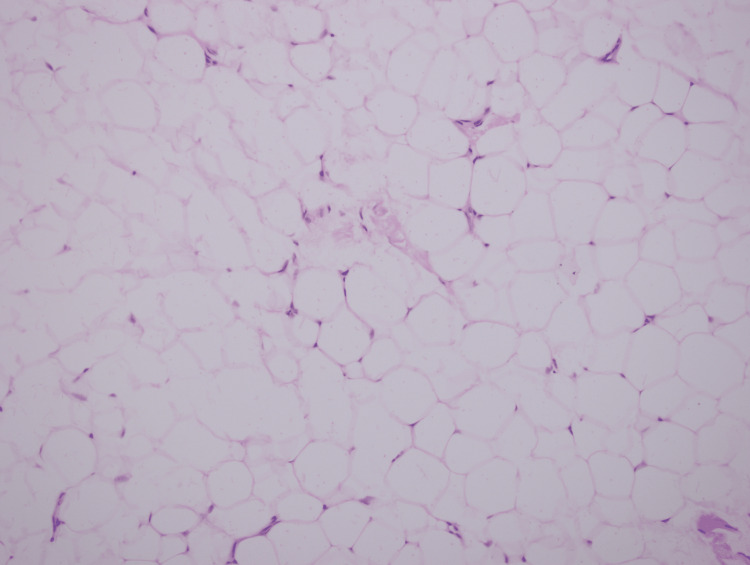
Low-power view (x20 magnification) histopathological image (hematoxylin and eosin) showing lobules of mature adipose cells representing a lipoma.

The clinical diagnosis and the management were discussed with the patient and afterward, the patient agreed to undergo surgical resection of the tumor. The surgery was conducted under general anesthesia using the right lateral cervical approach. Careful dissection was performed to expose the lesion, which appeared as a large yellowish mass. The lesion was excised completely. The recovery was uneventful. Histopathological examination showed lobules of mature adipose tissue, representing a lipoma (Figure 3).

Postoperatively, the patient had significant improvement in his swallowing. The patient was discharged on the fourth postoperative day. At the 4-week follow-up visit, the patient regained normal swallowing and experienced weight gain.

## Discussion

We report a rare case of a giant retropharyngeal lipoma that underwent successful surgical excision. The retropharyngeal space is a potential space in the neck that spans from the skull base to the mediastinum. It is bounded by the buccopharyngeal space anteriorly, and the prevertebral space posteriorly [3]. Primary tumors of the retropharyngeal space are uncommon.

We conducted a review of the literature published from 2000 to 2022, by searching PubMed, MEDLINE, and GoogleScholar databases. A total of 27 cases were identified, which are summarized in Table 1. The mean age of patients was 49.9 years with a range from 2 years to 81 years. Retropharyngeal lipoma was twice as common in men. The clinical presentations of patients with retropharyngeal lipomas varied widely. As in the present case, a significant number of patients presented with progressive dysphagia and unintentional weight loss. Notably, a considerable proportion of patients with retropharyngeal lipomas have a clinical picture of obstructive sleep apnea, with snoring and excessive daytime sleepiness, which was confirmed by polysomnography [2,4]. In only one case, the retropharyngeal lipoma was asymptomatic and was detected incidentally on imaging [5]. A retropharyngeal lipoma is often asymptomatic unless it reaches a considerable size to cause pressure effects on adjacent structures. In comparison with the reported cases, the present case has the largest size of retropharyngeal lipoma with a diameter of 13.8 cm.

**Table 1:** Review of reported cases of retropharyngeal lipoma in 2000–2022. CT: computed tomography; MRI: magnetic resonance imaging; N/A: not available.

Year	Author	Age	Gender	Clinical Presentation	Investigations	Size of Lipoma
2001	Senchenkov	49	Female	Dysphagia, snoring, nighttime awakening	Laryngoscopy, CT, MRI	8×5×4 cm
2001	Akhtar	76	Male	Dysphagia, cough, hoarseness	Barium swallow, CT	N/A
2002	Hockstein	64	Male	Obstructive sleep apnea	Laryngoscopy, CT, MRI	N/A
2004	Shivakumar	12	Male	Nasal obstruction, snoring, dysphagia	CT	3.8×2.6 cm
2005	Haddad	64	Female	Excessive daytime sleepiness, falls	Radiograph, CT	12×7×6 cm
2006	Gong	11	Female	Nasal obstruction, snoring, excessive daytime sleepiness	Laryngoscopy, radiograph, CT	8×4×2 cm
2006	Namyslowski	40	Male	Sleep disturbance	CT, laryngoscopy	11.7×2.2×4.5 cm
2007	Pillai	42	Male	Dyspnea	Radiograph, CT	8×5×11 cm
2007	Gupta	65	Male	Neck mass	CT	N/A
2007	Piccin	73	Female	Snoring, nasal congestion, hyponasal voice	CT, radiograph, nasopharyngoscopy	5×2×2.5 cm
2008	Huang	17	Male	Snoring, dysphagia, poor sleep quality, excessive daytime sleepiness	Nasopharyngoscopy, MRI	5×3×3 cm
2010	Sameer	35	Male	Neck swelling	Nasopharyngoscopy, CT	10×8 cm
2013	Lee	69	Female	Neck swelling, dysphagia, dyspnea	Nasopharyngoscopy, Ultrasound, CT, MRI	10×5×11 cm
2013	Chua	71	Male	Dysphagia	CT, MRI	9.4×6.7 cm
2014	Rangappa	75	Female	Neck swelling, dysphagia, dysphonia, dyspnea	Nasopharyngoscopy, CT	8×6 cm
2015	Ganakalyan	2	Male	Dysphagia	Nasopharyngoscopy, Radiograph, CT	3.98×4.7×7.0 cm
2015	Luczak	75	Male	Dysphagia, sleep apnea	Nasopharyngoscopy, CT	8.5×5.8×7.2 cm
2016	Kumar	48	Male	Dysphagia, neck pain	Barium swallow, radiograph, nasopharyngoscopy, MRI	9.5×3.8 cm
2016	Heaton	62	Female	Tongue pain, ear pain, dysphagia	Nasopharyngoscopy, MRI	4.4×2.3×1.4 cm
2017	Dilek	45	Male	Hoarseness, snoring, apnea	CT	N/A
2017	Leong	53	Male	Snoring, tiredness, throat pain	MRI	N/A
2018	Jin	10	Female	Snoring	CT, MRI	3.3×4.0 cm
2019	Ghamma	53	Male	Ptyalism, dysphagia	CT	7.3×2.6 cm
2020	Aydin	24	Male	Snoring, excessive daytime sleepiness, dyspnea, dysphagia	CT, MRI	12×7 cm
2020	Chysovitsiotis	64	Male	Snoring, hoarseness	CT, MRI	4.5×3×15 cm
2020	Ehlers	66	Female	Incidental finding	CT, MRI	N/A
2020	Bowers	81	Male	Dysphagia, weight loss	MRI	4.46×2.35 cm

Since the retropharyngeal space is not accessible for clinical inspection, cross-sectional imaging is vital to establish the diagnosis of retropharyngeal lipoma. On CT, the retropharyngeal lipoma appears as a well-circumscribed non-enhancing lesion with homogeneous fat attenuation [5]. Furthermore, MRI can further characterize the lesion and provides information on preoperative planning, and delineates the extension of the tumor [6]. The presence of post-contrast enhancement or internal septations may indicate the diagnosis of liposarcoma rather than lipoma [6]. The definite diagnosis of lipoma can be reached by histopathology. Hence, surgical resection is the treatment of choice and full recovery is expected. The resection can be performed by transoral or transcervical approaches. Transoral robotic surgery has also been used recently [7]. While the transoral approach is often preferred since it has lower morbidity, we performed the transcervical approach considering the giant size of retropharyngeal lipoma in the present case. Lipoma may have different histologic subtypes, including angiolipoma, chondrolipoma, and osteolipoma [8]. There is no evidence of malignant transformation or recurrence after complete resection of a retropharyngeal lipoma.

## Conclusions

Retropharyngeal space is a very rare location of lipoma. The case highlights the importance of considering retropharyngeal lesions when encountering a patient with progressive dysphagia. Since the retropharyngeal space is inaccessible to clinical inspection, cross-sectional imaging modalities are essential to make the diagnosis. Surgical resection is curative and the transcervical approach is safe and feasible for the resection of a giant retropharyngeal lipoma.
